# Diabetes, an independent poor prognostic factor of non-B non-C hepatocellular carcinoma, correlates with dihydropyrimidinase-like 3 promoter methylation

**DOI:** 10.1038/s41598-020-57883-1

**Published:** 2020-01-24

**Authors:** Satoko Umetsu, Hiroki Mizukami, Takeshi Saito, Chiaki Uchida, Akiko Igawa, Kazuhiro Kudo, Chieko Itabashi, Sho Osonoi, Guo Danyang, Takanori Sasaki, Soroku Yagihashi, Kenichi Hakamada

**Affiliations:** 1 Department of Pathology and Molecular Medicine, Hirosaki University Graduate School of Medicine, Hirosaki, Japan; 2 Department of Gastroenterological Surgery, Hirosaki University Graduate School of Medicine, Hirosaki, Japan

**Keywords:** Cancer, Genetics, Diseases, Endocrinology, Gastroenterology, Oncology, Pathogenesis

## Abstract

A concurrent increase in the prevalence of hepatocellular carcinoma (HCC) with that of type 2 diabetes (T2D) and obesity has been reported in the absence of hepatitis B virus surface antigen-negative/hepatitis C virus antibody-negative HCC (NBNC-HCC). However, the prognostic relevance of this association remains unclear. Promoter methylation (PM) of the dihydropyrimidinase-like 3 gene (*DPYSL3*) has been implicated in virus-related HCC. However, it remains unclear whether T2D influences PM in NBNC-HCC. We determined the influence of T2D on clinicopathological profile and PM of *DPYSL3* and *CDK2NA* in patients with NBNC-HCC who were divided into two groups: non-diabetes (non-DM; n = 46) and diabetes (DM; n = 47). DM was associated with a higher Union for International Cancer Control grade, marginal vascular invasion and tumour cell proliferation irrespective of the duration of T2D as well as higher rates of PM of *DPYSL3* than non-DM; however, PM of *CDK2NA* was similar between both groups. PM of *DPYSL3* reduced its expression which inversely correlated with reduced patient survival. In conclusion, T2D is associated with poor prognosis of NBNC-HCC in which a high frequency of PM of *DPYSL3* may play a pivotal role in its pathogenesis.

## Introduction

The rapid increase in the prevalence of type 2 diabetes mellitus (T2D) globally warrants an urgent need for improved disease prevention and management strategies^1–3^. Additionally, T2D is associated with an elevated risk of various cancers including hepatocellular carcinoma (HCC)^4^. Moreover, cancer is the leading cause of death in patients with T2D, with HCC being the most prevalent^5^.

HCC is the sixth most common neoplasm as well as the third leading cause of cancer-related death worldwide and fifth in Japan^6,7^. Viral hepatitis due to hepatitis B virus (HBV) and hepatitis C virus (HCV) infections was previously the leading cause of HCC. However, the number of patients with HCC in the absence of HBV surface antigen-negative/HCV antibody-negative HCC (NBNC-HCC) is rapidly increasing in Japan^8,9^, indicating that HCC developed independent of new HBV and HCV infections.

There is a close link between T2D and NBNC-HCC due to their association with non-alcoholic fatty liver disease (NAFLD) and obesity. More than 70% of individuals with T2D are estimated to have NAFLD^10,11^, of which 20% exhibit clinically relevant hepatic fibrosis, conferring an increased risk of progression to HCC^12,13^. While T2D and obesity elicit hepatic/peripheral insulin resistance, lipotoxicity, increased oxidative stress and chronic inflammation which result in the development of NBNC-HCC, the impact of T2D on the prognosis of NBNC-HCC has been partially evaluated^14^.

The molecular pathogenesis and progression of HCC involve multistep pathways encompassing the cooperation of genetic and epigenetic components at all stages of liver carcinogenesis^15^. In particular, promoter methylation (PM, an epigenetic DNA modification mechanism) is known to inactivate tumour suppressor genes, leading to the development of HCC^16^. Thus, the PM status of tumour suppressor genes can be related to the prognosis of HCC^17,18^. One such tumour suppressor gene is dihydropyrimidinase-like 3 (*DPYSL3*), a cell adhesion molecule expressed in the heart, brain and liver^19^. The expression of *DPYSL3* is downregulated by PM, which may contribute to metastasis in prostate and pancreatic cancers as well as poor prognosis of gastric cancer^20–22^. In HCC, the reduced expression level of *DPYSL3* through PM is inversely correlated with the expression of vascular endothelial growth factor (VEGF) and focal adhesion kinase (FAK), resulting in poor prognosis of HCC^23^.

Notably, PM is also implicated not only in the pathogenesis of T2D but also in cancer development complicated with T2D^24–26^. We previously demonstrated that enhanced PM of *CDH1* in long-term T2D is associated with poor prognosis of pancreatic ductal cancer (PDC)^26^. Therefore, we hypothesised that T2D similarly enhances PM of *DPYSL3* and deteriorates the prognosis of NBNC-HCC.

In this study, we evaluated the change in clinical outcome in NBNC-HCC confounded by T2D and the epigenetic modification of *DPYSL3*.

## Results

### Minimum impacts of T2D on clinicopathological changes in NBNC-HCC

The clinicopathological characteristics of the study patients are summarised in Table 1. The median age was 69 years (range, 48–82) in non-diabetic subjects (non-DM) and 67 years (range, 41–79) in diabetic subjects (DM). BMI was similar between non-DM (24.0; range, 19.5–32.7) and DM (23.7; range, 15.1–30.8). The median duration of diabetes in all patients was 9 years (range, 1–40). The pre- and post-surgery blood glucose and glycated haemoglobin (HbA1c) levels were significantly higher in DM than in non-DM (*p* < 0.01). The post-surgery elevated HbA1c levels (ΔHbA1c) were comparable between both groups. There were 22 patients (12 in DM and 10 in non-DM) with alcoholic liver disease (ALD) and 66 (32 in DM and 34 in non-DM) with a history of alcohol use. Transaminase, γ-GTP and tumour marker levels were comparable between both groups. Incidence of non-cancerous tumour of the liver was not significantly different between DM and non-DM. Recurrence after surgery was observed in 45 patients (24 in DM and 21 in non-DM), with 14 (8 in DM and 6 in non-DM) showing metastatic recurrence. There was no significant difference in terms of the site of recurrence between both groups. Pathological evaluation showed that the distribution of histological grade (wel and mod/por) was comparable between DM (41 and 6 cases, respectively) and non-DM (40 and 6 cases, respectively). There were no significant differences between the two groups in terms of capsule formation, capsule infiltration, septal formation and serosal invasion. Vascular invasion and tumour number had marginally, but not significantly, increased in DM compared with those in non-DM (*p* = 0.09 for vascular invasion and *p* = 0.07 for tumour number). The prevalence of high-grade tumour [Union for International Cancer control (UICC) stage II or III] was significantly higher in DM than in non-DM (*p* < 0.05).

**Table 1:** Clinical and pathological profiles of examined subjects.

	non-DM	DM	*p*-value
Number (male/female)	46 (36/10)	47 (42/5)	0.17
Age (years)	67 (41−79)	69 (48−82)	0.30
Body mass index	24.0 (19.5−32.7)	23.7 (15.1−30.8)	0.39
Duration of diabetes (years)		9.0 (1.0−40.0)	
HbA1c (NGSP, %) (pre-operation)	5.7 (5.1−6.2)	7.0 (5.5−11.4)	<0.01
HbA1c (NGSP, %) (post-operation)	5.7 (5.3−6.2)	6.3 (4.9−7.5)	<0.01
ΔHbA1c (NGSP, %) (pre-operation minus post-operation)	0.0 (−0.4–0.3)	1.8 (−1.0–5.3)	0.20
Diabetes therapy:			
Unknown		6.4% (3/47)	
Diet		27.7% (13/47)	
Oral hypoglycemic agent		48.9% (23/47)	
Insulin		17.0% (8/47)	
History of dyslipidemia	8.7% (4/46)	17.0% (8/47)	0.36
History of hypertension	43.5% (20/46)	57.5% (27/47)	0.22
Smoking habits (overall)	47.8% (22/46)	55.3% (26/47)	0.53
Smoking habits (Brinkman index ≧400)	30.4% (14/46)	38.3% (18/47)	0.51
Alcohol habit	71.7% (33/46)	68.1% (32/47)	0.20
AST (IU/L)	39.0 (18.0−136.0)	32.0 (13.0−193.0)	0.28
ALT (IU/L)	32.0 (15.0−131.0)	35.0 (8.0−157.0)	0.92
γ-GTP (IU/L)	89.5 (17−339)	78.0 (19−592)	0.38
AFP (ng/mL)	7.6 (1.2−24596)	7.1 (1.3−8209)	0.80
PIVKA-II (μg/mL)	90.0 (16.0−75000.0)	332.0 (15.0−60973.0)	0.16
Alcoholic liver	26.1% (12/46)	21.3% (10/47)	0.63
Background liver:			0.91
Normal liver	13.0% (6/46)	17.0% (8/47)	
Chronic hepatitis	60.9% (28/46)	59.6% (28/47)	
Cirrhosis	26.1% (12/46)	21.3% (10/47)	
Histological grade:			0.53
wel or mod	87.0% (40/46)	87.2% (41/47)	
por	13.0% (6/46)	12.8% (6/47)	
Growth type:			0.53
Expansive growth	87.0% (40/46)	85.1% (40/47)	
Invasive growth	8.7% (4/46)	14.9% (7/47)	
Formation of capsule	78.3% (36/46)	80.9% (38/47)	1.00
Infiltration to capsule	58.7% (27/46)	61.7% (29/47)	1.00
Septum formation	69.6% (32/46)	76.6% (36/47)	0.81
Serosal infiltration	4.3% (2/46)	6.4% (3/47)	1.00
Total vascular invasion:	30.4% (14/46)	51.1% (24/47)	0.09
Portal vein invasion	24.0% (11/46)	38.3% (18/47)	0.18
Hepatic vein invasion	19.6% (9/46)	25.3% (12/47)	0.62
Hepatic artery invasion	4.4% (2/46)	6.4% (3/47)	1.00
Biliary duct invasion	4.4% (2/46)	8.5% (4/47)	0.68
Tumor number	1.0 (1.0−2.0)	1.0 (1.0−5.0)	0.07
Tumor size (mm)	51.0 (10.0−180.0)	45.0 (12.0–180.0)	0.68
UICC stage(8^th^):			0.02
I	63% (29/46)	34% (16/47)	
II	33% (15/46)	55% (26/47)	
III	4% (2/46)	11% (5/47)	
Recurrence	46% (21/46)	51% (24/47)	0.68
Recurrence by metastasis	13% (6/46)	17% (8/47)	1.00

NGSP; National Glycohemoglobin Standardization Program, AST; Aspartate transaminase, ALT; Alanine transaminase, γ-GTP; γ-glutamyl transpeptidase, AFP; α-fetoprotein, PIVKA-II; protein induced by vitamin K absence or antagonist-II, wel; well-differentiated adenocarcinoma, mod; moderate-differentiated adenocarcinoma, por; poorly differentiated adenocarcinoma. Median(range).

### Accelerated PM of *DPYSL3* in T2D

Methylation-specific polymerase chain reaction (MSP) was performed using primers designed for *DPYSL3* and *CDK2NA* to examine methylation (M) and unmethylation (U) in promoter regions in 10 cases followed by DNA sequencing. A positive M band indicates significant methylation of the CpG region of the promoter region (Supplemental Fig. S1a). DNA sequencing of the MSP product confirmed methylation (Supplemental Fig. S1b) of cytosine, which was not converted to thymine. All CpG motifs in the promoter region of *DPYSL3* were methylated.

Based on previously confirmed accuracy and efficacy of MSP primers for *CDK2NA* in formalin-fixed, paraffin embedded (FFPE) specimens^26^, we found that the frequency of PM of *CDK2NA* in NBNC-HCC was not significantly different between DM (43%) and non-DM controls (33%) (*p* = 0.39) (Table 2). Similarly, the frequency of PM of *CDK2NA* in the non-cancerous tissue was not significantly different between DM (26%) and non-DM (11%) (*p* = 0.11). On the other hand, the frequency of PM of *DPYSL3* significantly increased in DM (77%) compared with that in non-DM (22%) in the cancerous tissue (*p* < 0.01). This pattern was also observed in the non-cancerous tissue wherein the frequency of PM of *DPYSL3* was significantly higher in DM (66%) than in non-DM (33%) (*p* < 0.01). Thus, *DPYSL3* was significantly methylated in DM compared with that in non-DM irrespective of the occurrence of NBNC-HCC.

**Table 2:** Promoter methylation analysis.

Gene	non-DM (n = 46)		DM (n = 47)	
	Tumor	Non-tumor	Tumor	Non-tumor
CDKN2A/P16	33% (15/46)	11% (5/46)	43% (20/47)	26% (12/47)
DPYSL3	22% (10/46)	33% (15/46)	77% (36/47)^*^	66% (31/47)^*^

*p < 0.01 vs non-DM.

### DPYSL3 and p16 expression evaluated by immunohistochemistry

**Figure 1:**
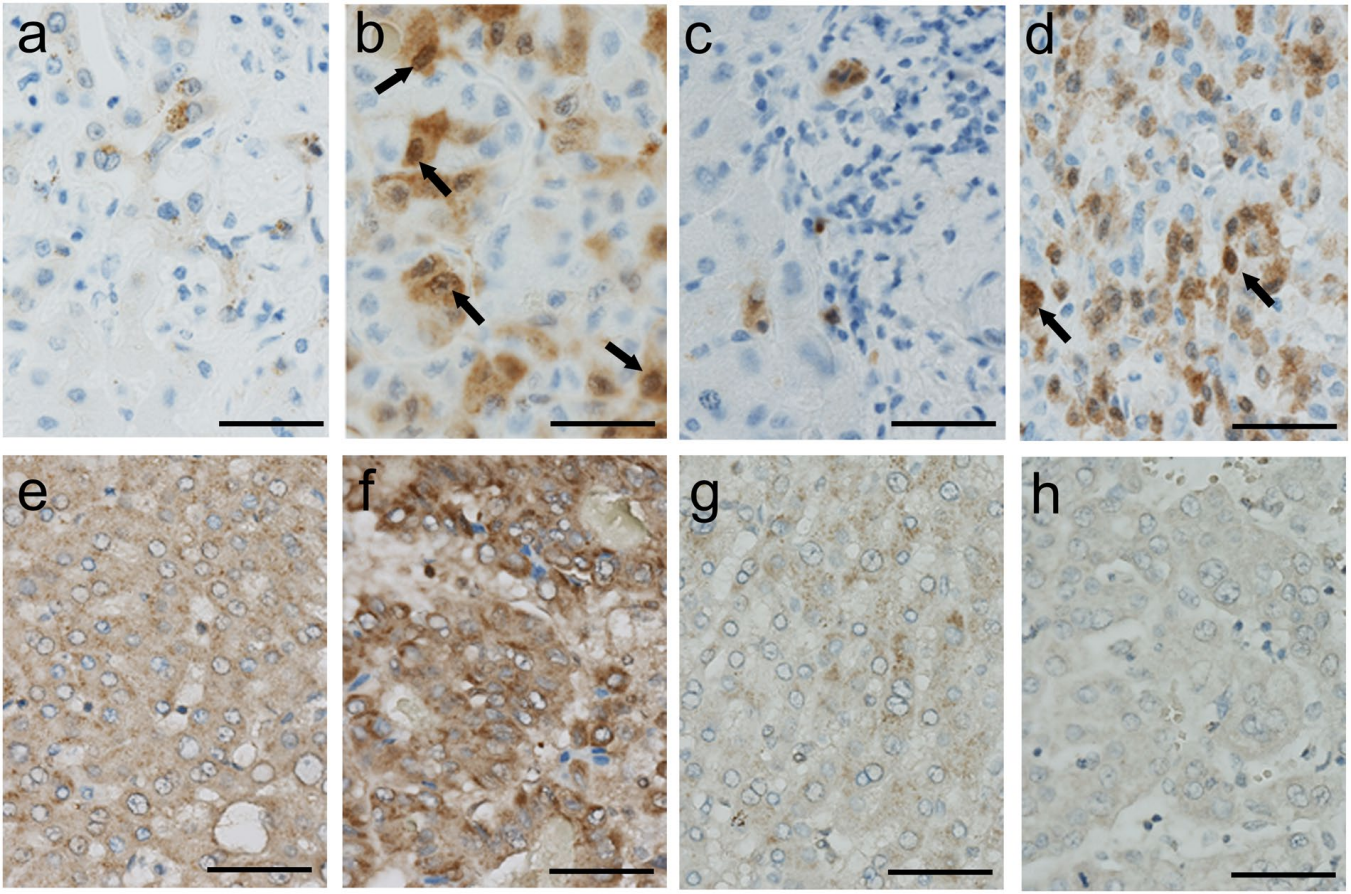
Expression of p16 and DPYSL3 in NBNC-HCC subjects. p16 expression (arrows) was sparse in the non-cancerous tissue (**a**) but was retained in the cancerous tissue (**b**) in non-DM. T2D failed to influence the expression of p16 in non-cancerous (**c**) and cancerous (**d**) tissues in NBNC-HCC. Diffuse expression of DPYSL3 was apparent in the non-cancerous (**e**) tissue and cancerous tissue (**f**) in non-DM, but its expression was attenuated in the non-cancerous (**g**) and cancerous (**h**) tissues in DM (scale bar = 50 μm).

**Figure 2:**
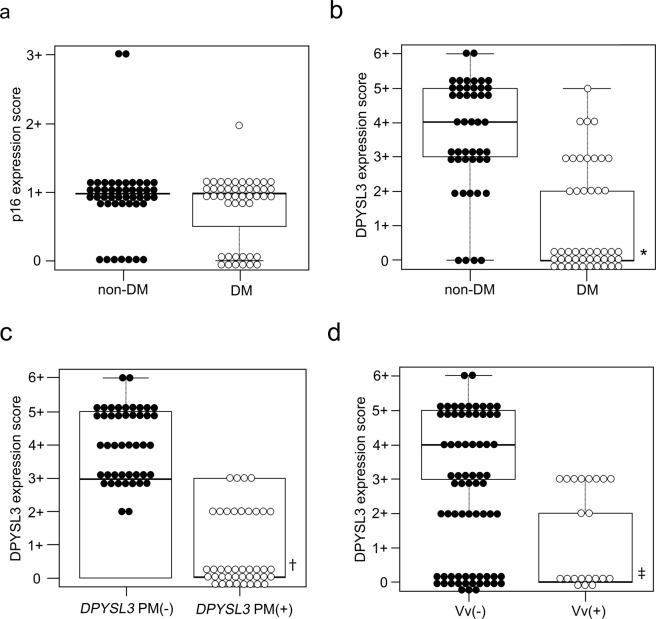
P16 and DPYSL3 expression score. p16 expression score in the cancerous tissue was comparable between non-DM and DM (**a**); Lower expression of DPYSL3 was evident in NBNC-HCC complicated with T2D (*p* < 0.01) (**b**); The expression of *DPYSL3* significantly reduced in *DPYSL3*-PM (+) compared with that in *DPYSL3*-PM (−) (*p* < 0.001) (**c**); the DPYSL3 expression score was attenuated in the hepatic vein invasion group (**d**). Vv; hepatic vein invasion. *p < 0.01 *vs* non-DM, ^†^*p* < 0.01 *vs* DPYSL3-PM (−), ^‡^*p* < 0.01 *vs* Vv (−).

Immunohistochemical evaluation revealed that the diagnosis of T2D did not affect the frequency of p16, coded by *CDK2NA*, cells labelled positive in non-cancerous and cancerous tissues in NBNC-HCC (Figs. 1a,d and 2a). Supporting this data, the expression score of Ki67 was comparable between non-DM and DM in the cancerous tissue (Supplemental Fig. S2). On the other hand, the expression of *DPYSL3* was preserved in both non-cancerous and cancerous tissues in non-DM controls, whereas its expression significantly reduced in DM (*p* < 0.01) (Figs. 1e–h and 2b). The protein expression score of DPYSL3 in cancerous tissue was irreversibly correlated with PM of *DPYSL3* (*p* < 0.01) (Fig. 2c), and it reduced in patients with NBNC-HCC showing hepatic vein invasion (Vv+) compared with that in patients showing non-venous invasion (Vv−) (*p* < 0.01) (Fig. 2d).

### Survival of patients with NBNC-HCC complicated with T2DM or PM of *DPYSL3*

Univariate analysis for disease-specific survival (DSS) showed that tumour multiplicity (multiple), serosal invasion, portal vein invasion, hepatic vein invasion, T2D history and PM of *DPYSL3* were the significant risk factors for reduced survival (Table 3). Smoking habit and history of metabolic disorders other than T2D such as hypertension and dyslipidaemia were not correlated with DSS (Table 3). Diabetes treatment was not correlated with DSS (Supplemental Table S1). Multivariate analysis further confirmed that hepatic vein invasion, tumour multiplicity (multiple), history of T2D and PM of *DPYSL3* remained the significant risk factors for reduced survival (Table 4).

**Table 3:** Univariate analysis of clinicopathological factors and disease-specific survival after resection of NBNC hepatocellular carcinoma (log-rank test).

Variable	Median DSS (month)	*p-*value
Age: <68 vs ≧68	56.0 vs 46.5	0.481
Male vs Female	43.0 vs 54.0	0.599
BMI: <23.9 vs ≧23.9	48.0 vs 52.0	0.453
Tumor multiplicity: single vs multiple	52.0 vs 40.5	0.0028
Tumor size (mm): <20 vs ≧20	59.0 vs 47.0	0.129
Tumor differentiation: wel, mod vs por	44.0 vs 42.5	0.333
Growth type: Expansive growth vs invasive growth	49.5 vs 51.5	0.963
Serosal infiltration: (−) vs (+)	53.0 vs 38.0	0.007
Formation of capsule: (−) vs (+)	69.5 vs 45.5	0.131
Infiltration to capsule: (−) vs (+)	59.0 vs 45.5	0.392
Septum formation: (−) vs (+)	59.0 vs 45.5	0.110
Bile duct invasion: (−) vs (+)	52.0 vs 51.0	0.697
Hepatic artery invasion:(−) vs (+)	51.0 vs 56.0	0.193
Portal vein invasion: (−) vs (+)	54.0 vs 43.0	0.007
Hepatic vein invasion: (−) vs (+)	56.5 vs 38.0	<0.001
T1-2 vs T3 (UICC)	51.5 vs 41.0	0.394
Alcoholic liver damage: (−) vs (+)	51.5 vs 82.25	0.841
Alcohol habit: (−) vs (+)	46.0 vs 54.0	0.243
History of T2D: (−) vs (+)	62.0 vs 41.0	0.002
HbA1c (%): <6.1 vs ≧6.1	50.5 vs 45.5	0.700
Blood glucose (mmol/L): <112.5 vs ≧112.5	60.0 vs 42.0	0.555
History of hypertension: (−) vs (+)	58.0 vs 46.0	0.832
History of dyslipidemia: (−) vs (+)	53.0 vs 44.0	0.644
Smoking habits (overall): (−) vs (+)	59.0 vs 45.0	0.726
Smoking habits (Brinkman index≧400): (−) vs (+)	54.0 vs 49.0	0.900
*DPYSL3* promoter methylation: (−) vs (+)	62.0 vs 43.5	0.009
*CDK2NA* promoter methylation: (−) vs (+)	54.0 vs 42.0	0.552

DSS; Disease-specific survival, BMI; body mass index, T2D; type2 diabetes, wel; well-differentiated adenocarcinoma, mod; moderate-differentiated adenocarcinoma, por; poorly differentiated adenocarcinoma.

**Table 4:** Multivariate analysis of clinicopathological factors and disease-specific survival after resection of NBNC hepatocellular carcinoma (Cox proportional hazards model).

Variable	Hazard ratio	95%CI	*p-*value
Hepatic vein invasion	2.489	1.128–5.493	0.024
Tumor multiplicity (multiple)	2.520	1.091–6.318	0.011
History of T2D	2.017	1.061–5.194	0.035
*DPYSL3* promoter methylation	2.656	1.210–6.483	0.024

95% CI; confidence interval, T2D; type2 diabetes.

Univariate analysis for overall survival (OS) showed that tumour multiplicity (multiple), serosal invasion, portal vein invasion, hepatic vein invasion, T3-4 cancer [UICC stage (8^th^)], history of T2D and PM of *DPYSL3* were the significant risk factors for reduced survival (Supplemental Table S2). However, smoking habit as well as a history of dyslipidaemia and hypertension was not correlated with OS (Supplemental Table S2). Diabetes treatment was also not correlated with OS (Supplementary Table S3). Multivariate analysis further confirmed that hepatic vein invasion, serosal invasion and history of T2D remained the significant risk factors for reduced survival (Supplemental Table S4).

**Figure 3:**
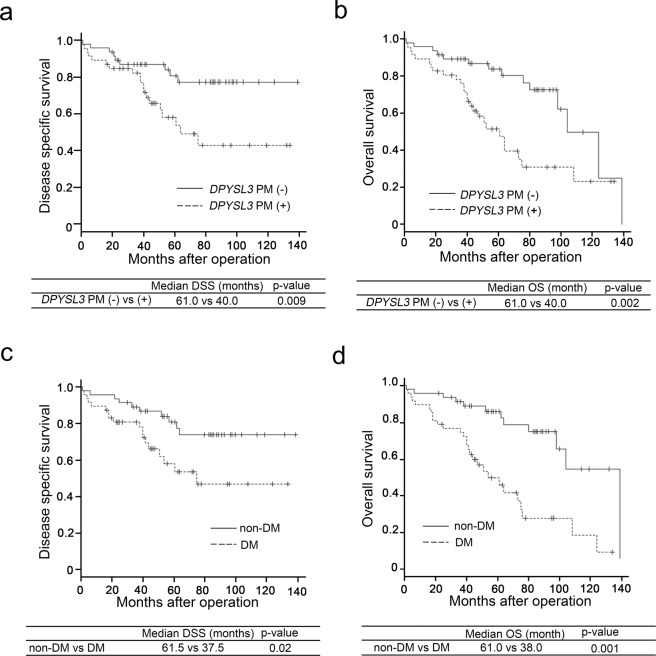
Survival curves based on disease-specific survival (DSS) and overall survival (OS). Results for survival curve of the *DPYSL3*-PM (+) group (fine break line) were significantly lower than those of the *DPYSL3*-PM (−) group (solid line) in terms of DSS (**a**) (*p* < 0.05) and OS (**b**) (*p* < 0.01). DM (fine break line) showed significantly worsened DSS (**c**) (*p* < 0.01) and OS (**d**) (*p* < 0.01) in NBNC-HCC than non-DM (solid line).

The Kaplan–Meier survival curve indicated a shortened DSS in DM compared with that in non-DM (Fig. 3a). OS also decreased in DM compared with that in non-DM (Fig. 3b). In addition, PM of *DPYSL3* reduced DSS and OS (Fig. 3c,d).

## Discussion

To the best of our knowledge, for the first time, we demonstrated that T2D had a negative impact on DSS and OS of patients with NBNC-HCC. The UICC stage of NBNC-HCC was significantly higher in DM than in non-DM. While DM exhibited enhanced PM of *DPYSL3* compared with non-DM, PM of *CDK2NA* was not influenced by T2D. The protein expression of DPYSL3 in tissues was inversely correlated with PM of *DPYSL3*, whose methylation status was also found to be an independent prognostic factor in multivariate analysis of DSS in patients with NBNC-HCC.

Metabolic disorders such as T2D and obesity are risk factors for HCC^4,12–14^. In this study, we identified that T2D was an independent prognostic factor for NBNC-HCC apart from known prognostic factors such as hepatic vein invasion, tumour multiplicity and epigenetic modifications involving PM of *DPYSL3*^18,27^. While a previous report showed significantly poorer long-term prognosis in obese patients with recurrent HCC mainly arising from viral hepatitis than in non-obese patients^28^, obesity showed minimum effects on the prognosis of NBNC-HCC in the present study. In parallel with our result, Nishikawa *et al*. showed that obesity did not affect survival in patients with NBNC-HCC after curative therapy^29^. Therefore, obesity may not be a stronger risk factor for worsening of the prognosis of NBNC-HCC than diabetes. Diabetes can exacerbate chronic inflammation, formation of advanced glycation end-products and aberrant insulin signalling, which trigger tumour formation and progression^30–32^. These indicate the importance of intervening for the improvement of lifestyle and of maintaining good glycaemic control, leading to the prevention of not only the onset of NBNC-HCC but also an unfavourable outcome.

Smoking habits are known to be a prognostic factor for HCC, whereas our results showed that only diabetes was correlated with reduced OS and DSS. Evans *et al*. showed that a significant increase in the risk of HCC was demonstrated among female smokers but not among male smokers^33^. Because our cohort was male dominant, no significant correlation was found between smoking habit and DSS/OS.

Long-term T2D (>3 years) was also a significant factor in the poor prognosis of PDC. In short-term T2D, tumour resection significantly lowered HbA1c level^26,34^, suggesting that short-term T2D is a transient diabetic state exerted by PDC. However, regardless of the duration, T2D was a poor prognostic factor for NBNC-HCC. We showed that ΔHbA1c remained unchanged after resection in NBNC-HCC, indicating its poor influence on glucose homeostasis. Therefore, T2D manifested in NBNC-HCC  is‘real’ diabetes which impacts the prognosis of NBNC-HCC similar to long-term T2D in PDC.

As described earlier, T2D deteriorated OS and DSS in PDC wherein long-term T2D was associated with a poor histological grade, vein invasion and a high frequency of PM of *CDH1*^26^. In contrast, except the UICC stage obtained in our study, the impact of T2D on each clinical and pathological characteristic of NBNC-HCC was minimum although the patients with T2D exhibited shorter DSS and OS than those without T2D. UICC stages are graded by tumour size (≤5 or >5 cm), tumour number (solitary or multiple), vascular invasion and direct invasion of adjacent organs^35^. The marginal, but non-significant, increase in tumour number and vascular invasion found in the presence of T2D suggest that the combined effect of these variables increases the UICC stage, leading to worse prognosis even if each variable has a small effect.

Suppression of DPYSL3 expression in HCC specimens was inversely correlated with a high expression of VEGF and FAK, which are associated with epithelial–mesenchymal transition (EMT)^23^. A hallmark of EMT is a change in cell morphology into spindle-shaped mesenchymal cells and notable downregulation of DPYSL3 which caused morphological changes into spindle- and fibroblast-like shape in a lung cancer cell line^36^, conferring these cells high invasion ability. This was borne out in NBNC-HCC complicated with T2D where low DPYSL3 expression enhanced hepatic vein invasion. However, regardless of T2D, tumour differentiation was comparable in patients with HCC accompanied by low DPYSL3 expression, corroborating previous findings showing increased capsule infiltration and vascular invasion^23^. These results suggest that EMT can partially be implicated in the poor prognosis of NBNC-HCC complicated with T2D and that mechanisms other than EMT regulate invasion in HCC complicated with PM of *DPYSL3*.

Although the prevalence of PM of *DPYSL3* did not increase in the non-cancerous tissue in a previous report^23^, we observed this characteristic in NBNC-HCC complicated with T2D. This may be ascribed to a strong potential of diabetes to elicit PM^24–26^, leading to an increase in tumour malignancy in NBNC-HCC cases with T2D.

On the other hand, *CDK2NA* is a common target of PM in HCC^37–39^. The frequency of *CDK2NA* PM-positive cases was lower in NBNC-HCC cases complicated with DM than in non-DM in our study. We attributed this to differential translational regulation of *CDK2NA* and *DPYSL3* because the prevalence and pattern of PM may be organ and carcinoma specific. Further, diabetes may modify the methylation pattern in each organ and carcinoma; therefore, it may be necessary to perform a comprehensive PM analysis using next-generation sequencing to characterise the PM status elicited by T2D in NBNC-HCC.

The progression of hepatic fibrosis and non-alcoholic steatohepatitis (NASH)/NAFLD score was not affected by T2D in this study, with the caveat being that NASH in patients with HCC was difficult to evaluate because of the need for its histological diagnosis. In end-stage NASH, the pathological characteristics can be lost and be, in effect, ‘burned out’ in which case a diagnosis of cryptogenic cirrhosis is made instead of NASH^40^. Thus, it has been acknowledged that a substantial proportion of patients with cryptogenic cirrhosis may actually have previously unrecognised NASH because patients with cryptogenic cirrhosis have a high prevalence of obesity and/or T2D^41–43^.

Treatment for T2D can also influence the onset of HCC and its associated mortality. While insulin and sulfonyl urea are associated with an increased risk of HCC, metformin treatment lowers this risk^44,45^. We found that metformin and insulin were not independent prognostic factors for DSS and OS in patients with NBNC-HCC. This might be attributed to the relatively small sample size (27 cases including 8 on metformin therapy and 10 on insulin therapy) in this study. We propose a future evaluation of T2D treatment effect with a larger number of cases.

NASH- and ALD-related HCC have different clinical course and prognosis, with the most inferior OS being identified in NASH-related HCC^46,47^. ALD-related HCC exhibited marginally high frequency of extra-hepatic malignancy compared with NASH-related HCC^48^. Because our cohort included patients with ALD-related HCC and those with non-ALD-related HCC, their distribution may have influenced the worse prognosis of diabetes. However, the ratio of patients with ALD-related HCC was comparable between non-DM and DM (26% vs 21%). Further, while OS and DSS were evaluated for different types of HCC including ALD-related HCC, no significant difference was identified among these types in this study; this was possibly because of the small number of ALD-related HCC cases (22/93, 23.7%). As another possibility, the aetiology in some non-ALD-related HCC cases was not associated with NASH because the pathological confirmation for NASH was not achieved in all non-ALD-related HCC cases due to the ‘burned out’ phenomenon. In addition to the ‘burned out’ phenomenon, because a significant ratio of our patients had a history of alcohol use, it is difficult to completely distinguish the aetiology of NASH- and ALD-related HCC.

This study has certain limitations. First, this study was a retrospective study conducted using only FFPE specimens; therefore, a future study should involve appropriate specimens including fresh specimens to examine dynamic glucose metabolism. Second, both alcoholic and non-alcoholic livers formed the study sample for NBNC-HCC, and it may be necessary to independently examine these groups. However, we based our design on a previous study which showed comparable pathological grading and parameters of HCC between alcoholic and non-alcoholic fatty livers^49^. Third, we investigated PM of only *CDK2NA* and *DPYSL3* in this study. Methylated genes other than *CDK2NA* and *DPYSL3* may be implicated in an unfavourable prognosis of NBNC-HCC complicated with T2D, particularly because a recent genome-wide study showed PM of several tumour suppresser genes implicated in the tumorigenesis of NBNC-HCC^50^. Nevertheless, the results of our study support evidence in favour of interventions to alleviate metabolic disorders for better prognosis of NBNC-HCC complicated with T2D. It is hoped that future studies will explore the possibility of developing effective demethylating agents for these diseases.

## Methods

### Patients

Between January 2005 and December 2016, patients who were diagnosed with NBNC-HCC and underwent initial hepatic resection at Hirosaki University Hospital, Aomori Prefectural Central Hospital, Hakodate Municipal Hospital and Hachinohe City Hospital were included in this retrospective study based on clinical data extracted from medical records. All investigations and experiments were performed after receiving the permission of the Ethical Committee of Hirosaki University Graduate School of Medicine (approved number #2017-162), and they were performed according to the guidelines of the Ethics Committee on Human Research Samples at the Japanese Society of Pathology. Informed consent was obtained from all participants and/or their legal guardians.

Among patients with HCC, those who were seronegative for HBVAg (HBsAg), HBVAb (HBsAb and HBcAb), and HCVAb without autoimmune liver disease, Wilson’s disease or hemochromatosis was considered to indicate NBNC-HCC. Diabetic patients with a history of hyperglycaemia fulfilled the criteria of diabetes proposed by the Japan Diabetes Society^51^. Patients with ALD were diagnosed based on habitual daily alcohol consumption of >40 g for men and >20 g for women and negativity for markers of HBV and HCV in their medical records. Hypertension was defined as blood pressure of ≥140/90 mm Hg or history of treatment for hypertension. Dyslipidaemia was defined as a total serum cholesterol level of ≥220 mg/dL, triglyceride level of ≥150 mg/dL or a prescription for dyslipidaemia. ‘Smoking habits (overall)’ was defined as a condition in which an individual continued to smoke within pre-surgery 1 year. A total of 94 assessed patients were screened and 93 were enrolled and split into a diabetic group (DM, 42 male/5 female) and non-diabetic group (non-DM, 36 male/10 female).

### Histopathological assessment

Histopathological assessment was independently performed using H&E-stained sections of each sample by three pathologists (HM, KK and CI). Pathological diagnosis of HCC was performed according to the 2010 WHO Classification of Tumours of the Digestive System, whereas staging was performed based on the 8^th^ edition of UICC^51^. Histological differentiation was divided into three stages according to cell and structural variants^52^: well-differentiated carcinoma (wel), moderately differentiated adenocarcinoma (mod) and poorly differentiated adenocarcinoma (por). When ≥2 tissue types and regions showing various degrees of differentiation were mixed, they were grouped according to the predominant tissue type and degree of differentiation.

### Genetic analysis

#### DNA extraction and bisulfite DNA modification

Tumour tissues without haemorrhage, necrosis or severe inflammation were selected. Non-tumour tissues adjacent to the tumour were selected. DNA extraction and bisulfite DNA modification were performed following the protocol reported previously^26^. In brief, DNA was extracted from FFPE sections (10-μm thickness) following the manufacturer’s instructions provided in the DNA extraction kit for FFPE (Qiagen K.K., Tokyo, Japan). Bisulfite DNA modification was conducted on the extracted samples with a commercially available kit (EpiTect Fast Bisulfite Conversion Kits, Qiagen K.K.). This process converts unmethylated cytosine residues to uracil while methylated cytosine residues remain unchanged^53^.

#### MSP

MSP was performed as previously described using primers for *DPYSL3*^54^ and *CDK2NA*^26^. The PCR product was electrophoresed on a 3% agarose gel. A positive methylated band (M) indicated high rates of methylation of the CpG region. PM of *DPYSL3* and *CDK2NA* was confirmed by Sanger sequencing^26^. MSP products were ligated using the TOPO cloning vector (Thermo Fisher Scientific K.K., Yokohama, Japan). After blue–white selection, a purified vector was digested with *Eco*RI. A 150-bp insert was sequenced using the ABI Prism 310 sequence analyser (Thermo Fisher Scientific K.K.) on the positive clone labelled using the VIC dye sequence kit (Thermo Fisher Scientific K.K.).

#### Immunohistochemical analysis

An automated immunohistochemistry instrument was applied for immunohistochemical analysis (Benchmark Ultra Automated Slide Preparation system, Ventana Medical Systems, Inc., Tucson, AZ, USA) as shown in our previous study^26^. Antibodies for p16 (Clone E6H4, pre-diluted, Ventana Medical Systems, Inc.) and DPYSL3 (Clone 1B8, LS-C133161, LifeSpan BioSciences, Seattle, WA, USA) were used. Staining intensity (score 0: no staining, 1: weaker staining than normal liver, 2: similar staining as normal liver and 3: stronger staining than normal liver) and staining range (score 0: no staining, 1: <30% staining, 2: 30%–69.9% staining and 3: >70% staining) were semiquantitatively scored. Further, the sum (minimum 0 to maximum 6) was graded as the degree of DPYSL3 expression. A total score of <4 indicated reduction in protein expression. p16 was evaluated as previously described by Saito *et al*.^26^.

#### Statistical analysis

All statistical analyses were performed with EZR (Saitama Medical Center, Jichi Medical University, Saitama, Japan) and the graphical user interface for R (The R Foundation for Statistical Computing, Vienna, Austria)^55^. This modified version of R commander was designed to add statistical functions frequently used in biostatistics. DSS was defined as the time between surgery and death from HCC. OS was calculated as the time between surgery and death from any cause. Continuous variables were analysed using non-parametric methods for non-normally distributed data (Mann –Whitney *U*-test) and are expressed as median (range). Categorical variables were analysed using the chi-squared test or Fisher’s exact test as appropriate and are expressed as number (percentage). Any variable with *p*-value of <0.05 on univariate analysis using the above test was considered a candidate for multivariate analysis using a Cox proportional hazards model. Survival curves were constructed using Kaplan–Meier analysis, and *p*-values were determined by the log-rank test for censored survival data. A *p*-value of <0.05 was considered statistically significant.

## Supplementary information

Supplementary Information.

## Data Availability

The datasets generated during the current study are available from the corresponding author on reasonable request.
