# Supplementary material for: Diabetes, an independent poor prognostic factor of non-B non-C hepatocellular carcinoma, correlates with dihydropyrimidinase-like 3 promoter methylation
Source: Sci Rep. 2020 Jan 24;10:1156. doi: 10.1038/s41598-020-57883-1 (PMC6981134; doi:10.1038/s41598-020-57883-1)
Supplement: Supplementary file 1 — Supplementary Information. [file 41598_2020_57883_MOESM1_ESM.pdf]

Diabetes, an independent poor prognostic factor of non-B non-C hepatocellular carcinoma, correlates with dihydropyrimidinase-like 3 promoter methylation

Satoko Umetsu[1,2], Hiroki Mizukami[1*], Takeshi Saito[1,2], Chiaki Uchida[1,2], Akiko Igawa[1,2], Kazuhiro Kudo[1], Chieko Itabashi[1], Sho Osonoi[1], Guo Danyang[1], Takanori Sasaki[1], Soroku Yagihashi[1] and Kenichi Hakamada[2]

[1]Department of Pathology and Molecular Medicine, Hirosaki University Graduate School of Medicine, Hirosaki, Japan

[2]Department of Gastroenterological Surgery, Hirosaki University Graduate School of Medicine, Hirosaki, Japan,

*Correspondence to Dr. H Mizukami, Department of Pathology and Molecular Medicine, Hirosaki University Graduate School of Medicine,

5 Zaifu-cho, Hirosaki, 036-8562 Japan

Phone 81-172-39-5025, Fax 81-172-39-5026, E-mail; hirokim@hirosaki-u.ac.jp

Running Title; unfavorable outcome of non-B non-C hepatocellular carcinoma in diabetes

Main text: 3681 words

Disclosure: There is no conflict of interest in any of the listed authors

This study was supported in part by KAKENHI (Grants-in-Aid for Scientific Research) from the Japanese Ministry of Education, Culture, Sports, Science and Technology to HM (#18K08462)

Supplemental Table S1. Univariate analysis of diabetes therapeutic factors and disease-specific survival after resection of NBNC hepatocellular carcinoma in diabetic patients (log-rank test).

Variable	Median DSS (month)	*P* value
**Diet therapy: (-) vs (+)**	42.0 vs 45.5	0.676
**Oral hypoglycemic agents: (-) vs (+)**	44.0 vs 43.0	0.965
**Metoformin: (-) vs (+)**	41.0 vs 52.5	0.297
**Insulin: (-) vs (+)**	45.5 vs 37.0	0.308

Supplemental Table S2. Univariate analysis of clinicopathological factors and overall survival after resection of NBNC hepatocellular carcinoma (log-rank test).

Variable	Median OS (month)	*P* value
**Age: ＜68 vs ≧68**	56.5 vs 47.0	0.514
**Male vs Female**	56.0 vs 47.5	0.582
**BMI: ＜23.9 vs ≧23.9**	48.0 vs 52.0	0.479
**Tumor multiplicity: single vs multiple**	52.0 vs 45.5	0.015
**Tumor size (mm):<20 vs ≧20**	59.0 vs 47.5	0.304
**Tumor differentiation: wel, mod vs por**	47.5 vs 53.0	0.105
**Growth type: Expansive growth vs invasive growth**	51.5 vs 46.0	0.976
**Serosal infiltration: (-) vs (+)**	53.0 vs 38.0	0.023
**Formation of capsule: (-) vs (+)**	69.5 vs 45.5	0.022
**Infiltration to capsule: (-) vs (+)**	59.0 vs 45.5	0.390
**Septum formation: (-) vs (+)**	59.0 vs 46.5	0.112
**Bile duct invasion: (-) vs (+)**	51.0 vs 52.0	0.787
**Portal vein invasion: (-) vs (+)**	54.0 vs 44.0	0.005
**Hepatic vein invasion: (-) vs (+)**	56.5 vs 43.0	0.001
**T1-2 vs T3-4 (UICC)**	51.5 vs 41.0	0.404
**Alcoholic liver damage: (-) vs (+)**	51.5 vs 52.5	0.832
**Alcohol habit: (-) vs (+)**	46.0 vs 54.0	0.228
**History of T2DM: (-) vs (+)**	62.0 vs 43.0	0.007
**HbA1c (%): <6.1 vs ≧6.1**	50.5 vs 47.0	0.745
**Blood glucose (mmol/L): <112.5 vs ≧112.5**	60.0 vs 43.0	0.074
**History of hypertension: (-) vs (+)**	98.9 vs 73.0	0.092
**History of dyslipidemia: (-) vs (+)**	98.0 vs 47.0	0.083
**Smoking habits (overall): (-) vs (+)**	104.0 vs 98.0	0.947

Supplemental Table S2. continued

**Smoking habits (Brinkman index≧400): (-) vs (+)**	104.0 vs 98.0	0.901
**DPYSL3 promoter methylation: (-) vs (+)**	62.0 vs 45.5	0.009
**CDK2NA promoter methylation: (-) vs (+)**	54.0 vs 43.0	0.552

OS; Overall survival, BMI; body mass index, T2DM; type2 diabetes, wel; well-differentiated adenocarcinoma, mod; moderate-differentiated adenocarcinoma, por; poorly-differentiated adenocarcinoma.

Supplemental Table S3. Univariate analysis of diabetes therapeutic factors and overall survival after resection of NBNC hepatocellular carcinoma in diabetic patients (log-rank test).

Variable	Median DSS (month)	*P* value
**Diet therapy: (-) vs (+)**	64.0 vs 51.0	0.98
**Oral hypoglycemic agents: (-) vs (+)**	47.0 vs 75.0	0.474
**Metoformin: (-) vs (+)**	61.0 vs 76.0	0.206
**Insulin: (-) vs (+)**	61.0 vs 36.0	0.788

Supplemental Table S4. Multivariate analysis of clinicopathological factors and overall survival after resection of NBNC hepatocellular carcinoma (Cox proportional hazards model)

Variable	Hazard ratio	95%CI	*P* value
**Hepatic vein invasion**	2.872	1.301－6.338	0.009
**Serosal infiltration**	2.482	1.076－5.723	0.033
**History of T2DM**	2.221	1.032－5.210	0.045

95% CI; confidence interval, T2DM; type2 diabetes.

Supplemantal Figure S1.

a

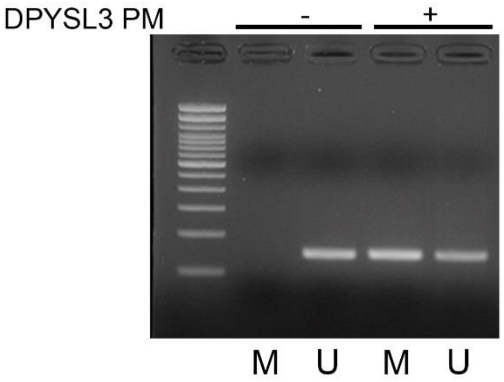

b

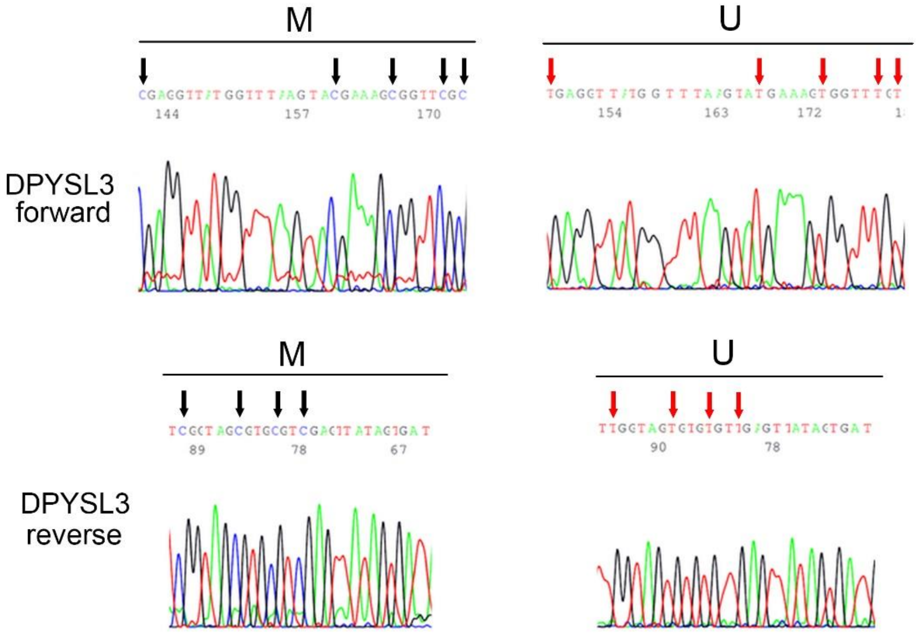

Supplemental Figure S2.

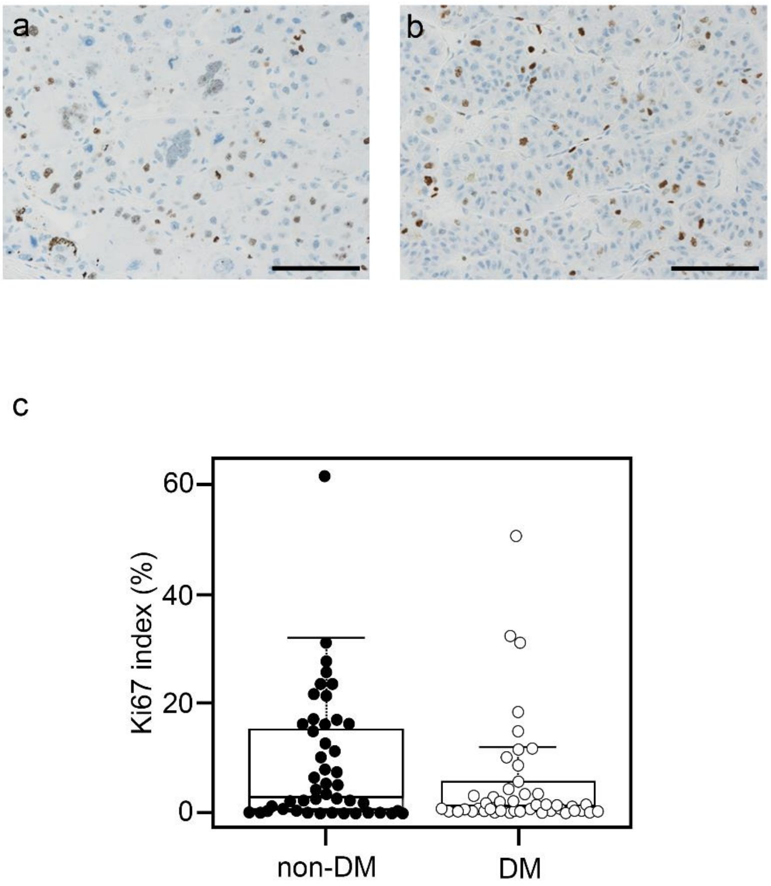

Legends for supplemental figures

Supplemental Figure S1.

Analysis by methylation-specific PCR in NBNC-HCC.

The subjects bearing promoter methylation (PM) of DPYSL3 showed methylated and unmethylated bands (**a**). Non-methylated tumor exhibited only unmethylated band. U = unmethylated, M = methylated. Sequencing of the PCR amplicons indicated methylated base of cytosine as C (black arrows), whereas C in unmethylated DNA was converted to thymidine (T) after bisulfide treatment (red arrows) (**b**). Lines under nucleotide sequence indicate primer sequence.

Supplemental Figure S2.

Cell proliferation analysis evaluated by Ki67 immunostaining

Ki67 was positive in the nucleus of hepatocellular carcinoma cells in both non-DM cases (**a**) and DM cases (**b**). Ki67 index was comparable between non-DM and DM (**c**). Bar represents 50 µm in each.
